# Unlocking the nutritional potential of chickpea: strategies for biofortification and enhanced multinutrient quality

**DOI:** 10.3389/fpls.2024.1391496

**Published:** 2024-06-07

**Authors:** Uday Chand Jha, Harsh Nayyar, Mahender Thudi, Radha Beena, P. V. Vara Prasad, Kadambot H. M. Siddique

**Affiliations:** ^1^ Indian Council of Agricultural Research (ICAR) – Indian Institute of Pulses Research (IIPR), Kanpur, Uttar Pradesh, India; ^2^ Department of Agronomy, Feed the Future Innovation Lab for Collaborative Research on Sustainable Intensification, Kansas State University, Manhattan, KS, United States; ^3^ Department of Botany, Panjab University, Chandigarh, India; ^4^ College of Agriculture, Family Sciences and Technology, Fort Valley State University, Fort Valley, GA, United States; ^5^ Department of Plant Physiology, College of Agriculture, Vellayani, Kerala Agriculture University, Thiruvananthapuram, Kerala, India; ^6^ Institute of Agriculture, The University of Western Australia, Perth, Australia

**Keywords:** chickpea, protein, carbohydrate, fat, quality traits

## Abstract

Chickpea (*Cicer arietinum* L.) is a vital grain legume, offering an excellent balance of protein, carbohydrates, fats, fiber, essential micronutrients, and vitamins that can contribute to addressing the global population’s increasing food and nutritional demands. Chickpea protein offers a balanced source of amino acids with high bioavailability. Moreover, due to its balanced nutrients and affordable price, chickpea is an excellent alternative to animal protein, offering a formidable tool for combating hidden hunger and malnutrition, particularly prevalent in low-income countries. This review examines chickpea’s nutritional profile, encompassing protein, amino acids, carbohydrates, fatty acids, micronutrients, vitamins, antioxidant properties, and bioactive compounds of significance in health and pharmaceutical domains. Emphasis is placed on incorporating chickpeas into diets for their myriad health benefits and nutritional richness, aimed at enhancing human protein and micronutrient nutrition. We discuss advances in plant breeding and genomics that have facilitated the discovery of diverse genotypes and key genomic variants/regions/quantitative trait loci contributing to enhanced macro- and micronutrient contents and other quality parameters. Furthermore, we explore the potential of innovative breeding tools such as CRISPR/Cas9 in enhancing chickpea’s nutritional profile. Envisioning chickpea as a nutritionally smart crop, we endeavor to safeguard food security, combat hunger and malnutrition, and promote dietary diversity within sustainable agrifood systems.

## Introduction

Chickpea (*Cicer arietinum* L.), a nutritionally dense pulse crop, is widely consumed by humans and cultivated annually, predominantly in semiarid and temperate climates under rainfed conditions (Gao et al., 2015). Global chickpea cultivation spans 14.56 million hectares (Mha), producing approximately 15 million tons (Mt) annually (FAOSTAT, 2021). Major producers include India, Australia, Pakistan, Central America, and East Africa (Knights and Hobson, 2016), with India leading production at 11.4 Mt from 9.9 Mha (FAOSTAT, 2021). Chickpea varieties are categorized as ‘desi’ and ‘kabuli’ based on seed shape and color (Knights and Hobson, 2016), with desi types predominant in Australia, Central America, East Africa, and India, while kabuli types thrive in the Mediterranean, Middle East, North Africa, and North America (Knights and Hobson, 2016; Grasso et al., 2022).

Global climate change and burgeoning human populations threaten food and nutritional security. Despite increased agricultural productivity, over 820 million people globally suffer from food insecurity, and at least 2 billion face nutritional insecurity (Jha et al., 2024). Approximately 3 billion people in Asia, Africa, and Latin America face micronutrient deficiencies, particularly zinc (Zn) and iron (Fe) (Welch and Graham, 2004; Darnton-Hill et al., 2006), crucial for optimal growth and development (Brown et al., 2002; Welch, 2002). Chickpea, inherently abundant in these micronutrients and vitamins, can help address ‘hidden hunger,’ particularly among infants and women of childbearing age in low-income countries (Broughton et al., 2003; Ufaz and Galili, 2008; Burchi et al., 2011). Moreover, chickpea contributes to human disease prevention, including diabetes, hyperlipidemia, kwashiorkor, and anemia (Younis et al., 2015).

**Figure 1:**
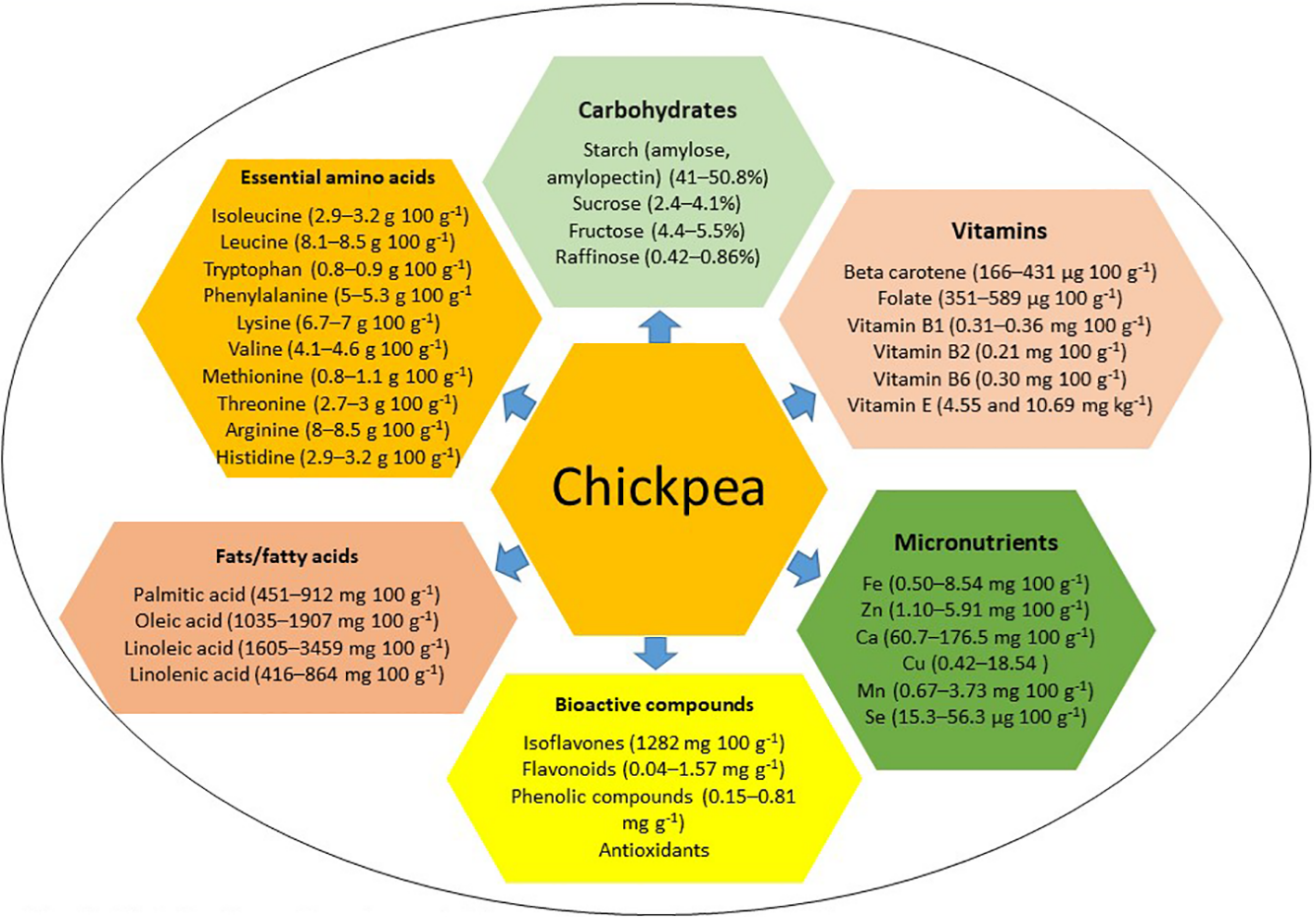
Distribution of major nutritional components in chickpea.

Chickpea, characterized by high dietary seed protein, abundant non-starch polysaccharides, low calorie content, low allergenicity, and high digestibility, offers a cost-effective protein source for low-income individuals and vegetarians (Kaur and Prasad, 2021; Yegrem, 2021; Begum et al., 2023). However, it lacks sulfur-containing essential amino acids methionine and cysteine (Jukanti et al., 2012; Grasso et al., 2022). Chickpea seeds are rich in carbohydrates, starch, fat, fiber, vitamins, and essential micronutrients (Heiras-Palazuelos et al., 2013; Kaur et al., 2019; Sharma et al., 2021; Grasso et al., 2022; Roorkiwal et al., 2022; Salaria et al., 2023) (see **Figure 1**). In Western countries, chickpea is mainly consumed in the form of ‘hummus’ (Wallace et al., 2016), with four tablespoons of chickpea-based hummus per day providing the equivalent of 2 cups of legumes per week and approximately 25 g of dietary fiber (Wallace et al., 2016). Approximately 1/3 cup of chickpea provides 5.4 g protein, 1.6 g fat, 16.7 g carbohydrates, 4.6 g dietary fiber, 29.9 mg calcium, 1.8 mg iron, 29.3 mg magnesium, 177.4 mg potassium, 104.9 µg folate, 0.6 mg vitamin A, and 0.2 mg vitamin C (Didinger and Thompson, 2021). Thus, regular chickpea consumption can meet the recommended daily allowance of secondary macronutrients and micronutrients (Thavarajah and Thavarajah, 2012). Chickpea can be used to make gluten-free bread (GFB), comprising 75% chickpea flour blended with 25% potato or cassava starch, for individuals with gluten-related disorders, enhancing nutritional quality, including dietary fiber (Santos et al., 2018). Moreover, chickpea harbors bioactive compounds like sterols, phenols, carotenoids, tannins, and isoflavones, with antioxidant properties and potential anti-glycemic and anti-cancer properties (Zhao et al., 2009; Tarzi et al., 2012; Rachwa-Rosiak et al., 2015; Domínguez et al., 2016; Wallace et al., 2016; Domínguez-Arispuro et al., 2018; Kaur and Prasad, 2021; Arora et al., 2023; Devi et al., 2023; Xiao et al., 2023). Recognizing that staple cereals alone cannot meet diverse micronutrient needs, supplementation with grain legumes like chickpea can provide essential micronutrients, fiber, and low glycemic index foods to combat nutritional insecurity and other health-related problems, particularly diabetes and obesity (Rebello et al., 2014; Ramani et al., 2021; Arya and Kumar, 2022; Gupta et al., 2023a). This review examines chickpea’s nutritional components, genetic determinants, and genomic regions contributing to improved nutrition. It also explores how emerging breeding tools and the CRISPR/Cas9 approach could enhance chickpea biofortification to help sustain global food security, address micronutrient deficiencies, mitigate malnutrition, and diversify food resources while supporting modern cropping systems and agricultural sustainability.

## Harnessing genetic variability for improving seed protein

Chickpea contains high-quality protein with exceptional bioavailability (Sharma et al., 2016; Kaur et al., 2019; Kumar et al., 2021), making them a potential solution for addressing protein malnutrition in developing countries by providing a high protein percentage (Iqbal et al., 2006). While breeders have made significant strides in developing over 200 commercially viable chickpea cultivars with improved yield and resistance to biotic and abiotic stresses (Grewal et al., 2023), the focus on quality improvement, particularly seed protein content (SPC), has been somewhat overlooked. Consequently, information on SPC across different chickpea cultivars remains incomplete, necessitating focused breeding efforts and genomics research to develop superior chickpea genotypes capable of addressing the mounting challenges of malnutrition. Research on genetic variability has uncovered a wide spectrum of SPC in chickpea. Singh et al. (2010) noted SPC ranging from 15.7–31.5%, while Özer et al. (2010) reported 17.55–23.31% in 91 kabuli chickpea genotypes, highlighting high-protein genotypes like Hatay2 (23.3%), Hatay3 (23.9%), and Mersin 4 (23.23%). Gaur et al. (2016) explored the genetic control of SPC in an ICC5912 × ICC17109 F_2_ population, reporting substantial genetic variability in pink-flowered segregants (12.84–26.72%), blue-flowered segregants (17.10–26.08%), and white-flowered segregants (14.77–24.10%). Phenotypic assessments across diverse chickpea germplasm indicated significant genetic variability for SPC, ranging from 15.6–22.4% (Sharma et al., 2013; Misra et al., 2016; Upadhyaya et al., 2016b), 4.6–33.9% (Bhagyawant et al., 2018), 16.56–24.64% (Roorkiwal et al., 2022), 16.3–26.2% (Srungarapu et al., 2022a), and 20–24.8% (Onder et al., 2023). Similarly, Samineni et al. (2022) assessed SPC and micronutrients in 140 chickpea genotypes, revealing variability ranging from 11.6–24.8% under non-stress conditions, 15.7–26.2% under drought stress, and 15.9–24.7% under heat stress. A comprehensive analysis of 402 chickpea genotypes at two locations in India (Ludhiana and New Delhi) indicated SPC variability ranging from 18.19–33.56%, with the highest values in GNG 2144 (28.81%), H 82-2 (28.29%), Pusa 5023 (26.28%), and Pusa 417 (26.00%) (Grewal et al., 2023). Interestingly, protein contents among commercially cultivated varieties ranged from 18.19–30.44%, relatively lower than germplasm accessions (19.41–33.56%) (Grewal et al., 2023). Farida Traore et al. (2022) reported broad variability in SPC (18.9–32.4%) in 282 advanced chickpea breeding lines in Morocco. In another study, SPC varied between desi and kabuli types, with desi types generally lower (20.29%) than kabuli types (24.51%) (Sharma et al., 2013; Ghribi et al., 2015). Comparative studies have also revealed differences in SPC between kabuli (23.15–25.97%), desi (23.19–28.0%), and wild species (23.03–26.82%) (see **Supplementary Table S1**), with one study identifying GL 12021 and GNG 2171 (desi), L552 (kabuli), and *C. pinnatifidum* ILWC 261 (wild) as high-protein lines (Kaur et al., 2019).

Chickpea wild relatives are valuable reservoirs for agriculturally significant traits, including SPC (Ocampo et al., 1998; Sharma et al., 2021). Ocampo et al. (1998) assessed 228 chickpea accessions from eight annual wild species, reporting significant genetic variability for SPC, ranging from 16.8% (*C. cuneatum*) to 26.8% (*C. pinnatifidum*). Of the wild species, *C. yamashitae* exhibited the highest SPC (21.7%), while *C. echinospermum* had the lowest (19.2%) (Ocampo et al., 1998) (see **Supplementary Table S1**). A subsequent analysis of 41 cultivated chickpea accessions and the eight annual wild *Cicer* species unveiled significant variation in SPC (21–25.5%), with the highest in ICC17141 (*C. chorassanicum*), followed by ICC17124 (*C. reticulatum*; 25.1%), ICC17262 and ICC17261 (both *C. reticulatum*; 24.5%), and ICC20236 (*C. chorassanicum*; 24.3%) (Sharma et al., 2021). Despite identifying numerous chickpea genotypes with high protein content, it has been found that grain yield negatively correlates with seed protein (Srungarapu et al., 2022b), indicating a tradeoff between protein content, seed size, and grain yield (Gaur et al., 2016). Nonetheless, selecting transgressive segregants with high SPC and moderate grain yield holds promise for developing chickpea cultivars with improved SPC (Gaur et al., 2016). Commercially released cultivars with high SPC, viz., GNG 2144, H-82-2, L 144, Pusa 112, and Pusa 5023 (Grewal et al., 2023), could be potential donors in future chickpea breeding programs aimed at enhancing SPC content and yield.

Chickpea proteins mainly comprise albumin (8–12%), globulin (53–60%), prolamin (3–7%), and glutelin (19–25%) (Day, 2013; Grasso et al., 2022), with significant variability observed between desi and kabuli types. Chickpea flour exhibits elevated levels of essential amino acids (39.89 g 100 g^–1^ protein) compared to wheat flour (32.20 g 100 g^–1^ protein), including arginine, aspartic acid, and glutamic acid, with a combined 36.85 g 100 g^–1^ protein for kabuli and 34.53 g 100 g^–1^ protein for desi varieties (Singh et al., 2010; Begum et al., 2023). Notably, chickpea contains essential amino acids above the recommended amounts by the WHO, including lysine (6.2–6.7 g 16 g^–1^ N; recommended 1.8), isoleucine (3.1–4.0 g 16 g^–1^ N; recommended 1.5), leucine (6.5–7.1 g 16 g^–1^ N; recommended 2.1), phenylalanine (5.1–5.8 g 16 g^–1^ N; recommended 2.1), threonine (3.1–3.4 g 16 g^–1^ N; recommended 1.1), valine (3.5–4.1 g 16 g^–1^ N; recommended 1.5), and histidine (2.3–2.8 g 16 g^–1^ N; recommended 1.5) (Onder et al., 2023). Moreover, selected commercially released chickpea cultivars exhibited higher essential amino acid contents than the WHO recommendations (see **Table 1**) (Grewal et al., 2023). Desi types have higher methionine (1.4%) than kabuli types (1.1%) (see **Supplementary Table S1**), with notable differences also reported for leucine, lysine, and serine (Ghribi et al., 2015; Ramani et al., 2021). Grewal et al. (2023) reported significant positive associations between SPC and aspartic acid, isoleucine, and phenylalanine but negative associations with methionine and cysteine. Therefore, cultivars with high amino acid content could be used to develop future biofortified chickpea cultivars to combat protein deficiency malnutrition. Among the major chickpea proteins, albumins (water-soluble proteins) are rich in sulfur-containing amino acids like methionine and cysteine (Bhatty, 1982), while globulins are salt-soluble proteins (Osborne, 1924), with legumin and vicilin the major globulins in chickpea (Boye et al., 2010; Day, 2013). Legumin contains higher methionine and cysteine levels than vicilin (Shevkani et al., 2019). Glutelins are soluble in dilute acid or alkali and contain methionine and cysteine (Singh and Jambunathan, 1982). Prolamins (alcohol-soluble) feature a high proportion of proline and glutamine (Osborne, 1924; Rachwa-Rosiak et al., 2015).

**Table 1:** Genetic variability for seed protein/lipid/carbohydrate content in chickpea from different countries.

Nutrition	Number of genotypes used	Quantity	Country reported from	Reference
**Protein**	228	16.8–26.8%	Syria	Ocampo et al. (1998)
	91 kabuli	17.55% and 23.31%	Turkey	Özer et al. (2010)
	187 (desi and kabuli)	13.25–26.77%	India	Jadhav et al. (2015)
	336	15.6–22.4%	India	Upadhyaya et al. (2016b)
	215	4.60–33.90%	India	Bhagyawant et al. (2018)
	15 cultivated and 15 wild types	23–26%	India	Kaur et al. (2019)
	41 cultivated and 8 wild types	21–25.5%	India	Sharma et al. (2021)
	258	16.56–24.64%	India	Roorkiwal et al. (2022)
	280	16.3–26.2%	India	Srungarapu et al. (2022a)
	140	11.6–24.8%	India	Samineni et al. (2022)
	402	18.19–33.56%	India	Grewal et al. (2023)
	282 advanced breeding lines	18.9–32.4%	Morocco	
**Lipid/fat**	91 kabuli	4.45–6.11%	Turkey	Özer et al. (2010)
	14 genotypes	5.68–9.01%	Argentina	Marioli Nobile et al. (2013)
	256 accessions	Palmitic acid (451–912 mg 100 g^–1^)	Canada	Salaria et al. (2023)
		Linoleic acid (1605–3459 mg 100 g^–1^)		
		Alpha-linolenic acid (416–864 mg 100 g^–1^)		
		Oleic acid (1035–1907 mg 100 g^–1^)		
**Carbohydrates**	30 genotypes	Total starch (26.49–39.27%)	India	Kaur et al. (2019)
		Total sugars (27.34–54.6%)		
	211 genotypes	Sucrose (3.57–54.12 mg g^–1^)	India	Elango et al. (2022)
		Stachyose (2.77–59.43 mg g^–1^)		
		Raffinose (0.16–15.13 mg g^–1^)		

## Carbohydrate composition

Chickpea seed typically contains 50–58% carbohydrates (Jukanti et al., 2012), with starch being the primary component (41–50.8%) (Singh, 1985). Additionally, chickpea seed contains cellulose (4–13%), hemicellulose (3.5–8.8%), and pectin (1.5–3.8%) (Wood and Grusak, 2007). Kabuli and desi chickpea varieties contain amylose and amylopectin, with both types containing more amylopectin than amylose (Singh et al., 2004) and kabuli types typically exhibiting higher amylose contents than desi types (Grasso et al., 2022). Thus, chickpea starch has a low glycemic index, making it suitable for diabetic individuals (Kaur and Prasad, 2021). The characteristics of chickpea starch depend on factors like amylose content, swelling power, solubility, and water-binding capacity (Miao et al., 2009). Desi chickpea types typically exhibit total and apparent amylose contents of 35.24% and 31.11%, respectively, while kabuli types have 31.80% and 29.93%, respectively (Miao et al., 2009). Kabuli starch has a higher *M*_w_ (5.382×10^7^ g mol^–1^) than desi starch (3.536×10^7^ g mol^–1^) (Miao et al., 2009). Genetic variability in starch content among 91 kabuli chickpea landraces from Turkey ranged from 40.07–41.76% (Özer et al., 2010). An assessment of four selected chickpea genotypes revealed significant genetic variability for starch (30.6–49.9%), glucose (3.4–4.7%), fructose (4.4–5.5%), and sucrose (2.4–4.1%) (Ereifej et al., 2001). In addition to starch, chickpea carbohydrates comprise dietary fiber (18–22%), insoluble sugars (10–18% crude fiber), and soluble sugars (4–8%) (Tosh and Yada, 2010), with desi types exhibiting higher insoluble dietary fiber than kabuli types due to thicker seed coats (Rincon et al., 1998). Sánchez-Mata et al. (1998) reported various monosaccharide concentrations in chickpea, including ribose (0.11 g 100 g^–1^), galactose (0.7 g 100 g^–1^), glucose (0.05 g 100 g^–1^), and fructose (0.25 g 100 g^–1^). In one study, chickpea fiber ranged from 4.9–5.5% (Ereifej et al., 2001), while another reported 2.88% in chickpea genotypes originating from Sicily (Patane, 2006). In a study investigating soluble sugars, fructose content ranged from 3.66–4.33 mg g^–1^ in kabuli types, 2.0–5.33 mg g^–1^ in desi types, and 5.0–10.33 mg g^–1^ in wild chickpea species (Kaur et al., 2019). They also noted positive association of soluble sugar with starch content.

Sucrose, raffinose, verbascose, and stachyose form the primary soluble sugars present in chickpea seeds (Chibbar et al., 2010). Raffinose and stachyose are galactosyl derivatives of sucrose, with raffinose having one galactose moiety and stachyose having two galactose moieties attached to sucrose via α (1-6) glycosidic linkage (Chibbar et al., 2010; Kaur et al., 2019). These compounds are classified as raffinose family oligosaccharides (RFOs), which are non-digestible by humans and animals, often causing flatulence (Elango et al., 2022). Chickpea oligosaccharide levels varied among different cultivars, ranging from 5.54–8.82%, including 0.25–0.73% verbascose, 1.54–3.18% stachyose, 2.04–5.26% ciceritol, and 0.42–0.86% raffinose (Tosh and Yada, 2010; Singh and Kumari, 2011). Genetic variability for RFOs in chickpea has been noted, ranging from 10.33 mg g^–1^ in wild chickpea (accession EC 366342) to 0.66 mg g^–1^ in kabuli chickpea (GLK 14313) (Kaur et al., 2019). Taking into account the association of RFO with other sugars, RFO revealed a positive and significant correlation with raffinose, stachyose, and verbascose in both desi and kabuli types. Similarly, sucrose also exhibited a positive and significant correlation with raffinose, stachyose, and verbascose in both desi and kabuli types (Gangola et al., 2013).

## Lipid/fat/oil composition

Fatty acids are classified into saturated fatty acids, monounsaturated fatty acids (MUFAs), and polyunsaturated fatty acids (PUFAs). Chickpea oil predominantly comprises unsaturated fatty acids (Gul and Egesel, 2008). Among grain legumes, chickpea has the highest fat (6.04%) (Allen et al., 2006). The lipid profile of chickpea includes storage lipids, primarily triacylglycerols, and membrane lipids like phospholipids, sphingolipids, glycolipids, and phytosterols (Madurapperumage et al., 2021). Both triacylglycerols and membrane lipids contribute to chickpea’s overall fat content (Zia-Ul-Haq et al., 2007; Madurapperumage et al., 2021). Chickpea seeds typically contain 56–67% triacylglycerols and 17–20% phospholipids (Zia-Ul-Haq et al., 2007). The recommended daily dietary allowance for infants and adults is 13.5 g palmitic acid, 7.5 g linoleic acid, 1.2 g alpha-linolenic acid, and 24 g oleic acid (Ervin et al., 2004). Daily consumption of chickpeas can provide 19.5 g of unsaturated fatty acids (Wallace et al., 2016). Interestingly, uncooked chickpeas contain more polyunsaturated fatty acids (2.73 g 100 g^–1^) than cooked chickpeas (1.15 g 100 g^–1^). Additionally, chickpea consumption contributes to lowering the intake of saturated fatty acids (22.4 g day^–1^), total fats (76.4 g day^–1^), and cholesterol (227 mg day^–1^) (Wallace et al., 2016). Various studies have reported significant variation in grain fat, ranging from 4.45–7.1% (Jood et al., 1998; Ereifej et al., 2001; Ravi and Harte, 2009; Shad et al., 2009; Özer et al., 2010). For instance, Özer et al. (2010) reported 4.45–6.11% grain fat in 91 Turkish kabuli chickpea samples, while Jood et al. (1998) reported 6–6.33% grain fat in Indian chickpea genotypes.

Chickpea oil content ranges from 3.8–10% (Gul and Egesel, 2008; Jukanti et al., 2012). The prominent fatty acids in chickpea oil are linolenic, oleic, and palmitic acids, with linoleic acid (18:2) the most abundant, ranging from 52.36–57.98% (Gul and Egesel, 2008). Omega-6 linoleic fatty acid constitutes the majority of chickpea oil (46–62%), followed by omega-9 oleic acid (24.12–29.48%) (Wood and Grusak, 2007; Gul and Egesel, 2008) and palmitic acid (16:0) (10.86–12.38%) (Gul and Egesel, 2008). Analysis of 14 Argentinean chickpea genotypes revealed oil contents ranging from 5.68–9.01%, with genotypes G101, P39, and L9 containing the most linoleic acid (mean 58.73%), linolenic acid (mean 3.06%), and oleic acid (18:1 omega-9) (mean 43.01%), respectively (Marioli Nobile et al., 2013). A fatty acid composition analysis in 256 chickpea accessions revealed wide genetic variability for palmitic acid (451–912 mg 100 g^–1^), linoleic acid (1605–3459 mg 100 g^–1^), alpha-linolenic acid (416–864 mg 100 g^–1^), and oleic acid (1035–1907 mg 100 g^–1^) (Salaria et al., 2023). A correlation study conducted by the same research group revealed a significant negative correlation between palmitic acid and linoleic acid, as well as between alphalinolenic acid and oleic acid. Further research on fatty acid composition in wild chickpea species could enhance our understanding and facilitate the development of biofortified chickpea varieties with improved fatty acid profiles to address nutritional needs among the human population.

## Micronutrients

Chickpea seeds are a rich source of essential micronutrients like Fe, Zn, calcium (Ca), copper (Cu), and manganese (Mn) (Ibrikci et al., 2003; Jahan et al., 2019; Sharma et al., 2021; Roorkiwal et al., 2022; Srungarapu et al., 2022a). Feeding studies have indicated that daily chickpea consumption can provide 17.4 mg Fe (Wallace et al., 2016). Uncooked chickpea contains higher Zn (2.76 mg 100 g^–1^) than cooked chickpea (1.53 mg 100 g^–1^). Moreover, consuming 100 g of raw chickpeas can provide 160 mg Ca, 138 mg Mg, 4.1 mg Zn, and 5.0 mg Fe (Jukanti et al., 2012; Begum et al., 2023), highlighting the benefits of consuming uncooked chickpea for micronutrient uptake. The average Fe content in chickpea ranges from 3.0–14.3 mg 100 g^–1^ (Diapari et al., 2014). A recent study reported a wide range of genetic variability for grain Fe (0.50–8.54 mg 100 g^–1^) and Zn (1.10–5.91 mg 100 g^–1^) in 402 chickpea accessions across two locations, with Heera, H82-2, and H214 identified as promising genotypes for high Zn (>4 mg 100 g^–1^) and L550, KGD1168, PG114, JG74, and ICCV6 for high Fe (>6 mg 100 g^–1^) (Grewal et al., 2020). Samineni et al. (2022) investigated associations between marker traits and grain Fe and Zn contents in 140 chickpea accessions, revealing significant genetic variation for grain Fe (47.8–83.0, 49.4–86.2, and 41.4–77.6 mg kg^–1^ under non-stress, drought stress, and heat stress, respectively) and Zn (29.5–55.0, 28.1–63.1, 29.7–55.4 mg kg^–1^ under non-stress, drought stress, and heat stress, respectively). Similarly, Srungarapu et al. (2022a) noted wide genetic variability for grain Fe (44.1–76.7 mg kg^–1^) and Zn (36.3–56.2 mg kg^–1^) with high heritability (85–98%) in a two-year assessment of 280 chickpea accessions. However, grain Fe exhibited significant positive associations with Zn and 100-seed weight but a highly negative correlation with yield under all tested environments, consistent with findings by Samineni et al. (2022). Similarly, grain protein content negatively correlated with grain yield across all environments, suggesting that breeding programs should focus on selecting genotypes with moderate yield and high nutrient content, especially protein and micronutrients, rather than solely increasing nutrient content (Srungarapu et al., 2022a). Moreover, Roorkiwal et al. (2022) reported significant genetic variation in 258 chickpea accessions for various nutritional components: Fe (2.26–7.25 mg 100 g^–1^, mean: 4.36), Ca (60.7–176.5 mg 100 g^–1^, mean: 108.6), Mg (64.08–134.57 mg 100 g^–1^, mean: 100.35), Mn (0.67–3.73 mg 100 g^–1^, mean: 1.78), phytic acid (2.07–19.38 mg g^–1^, mean: 10.68), and Zn (1.15–4.59 mg 100 g^–1^, mean: 2.76. Similarly, Fayaz et al. (2022) reported significant genetic variation in various micronutrients in 147 chickpea genotypes over a two-year evaluation: Zn (1.45–20.49 and 0.936–20.58 mg 100 g^–1^, Fe (7.93–19.82 and 10.36–12.72 mg 100 g^–1^), Cu (0.42–18.54 and 0.366–19.03 mg 100 g^–1^), and Mn (0.907–5.43 and 0.48–12.28 mg 100 g^–1^) and Farida Traore et al. (2022) reported a wide range of genetic variability in 282 advanced chickpea breeding lines for Fe (3.12–8.1 mg 100 g^–1^) and Zn (3.21–8.61 mg 100 g^–1^). A correlation analysis revealed significant positive associations between grain protein content and Ca, Mn, Zn, β-carotene, and phytic acid contents but negative associations between β-carotene and Mn and between phytic acid and vitamin B1 (Roorkiwal et al., 2022). Another study reported that grain protein content negatively correlated with Zn content (Farida Traore et al. (2022), with Fe and Mn content (Sharma et al., 2021). These findings suggest an opportunity to target nutrient improvement in chickpea breeding programs.

A comprehensive analysis of 41 cultivated chickpea accessions and eight annual wild Cicer species revealed significant variation in Fe (48.6–166 mg kg^–1^) and Zn (35.3–47 mg kg^–1^) in six wild Cicer species—*C. reticulatum*, *C. echinospermum*, *C. bijugum*, *C. pinnatifidum, C. chorassanicum*, and *C. yamashitae*—compared to cultivated chickpeas (∼42 mg kg^–1^ for Fe and ∼28 mg kg^–1^ for Zn) (Sharma et al., 2021). The wild species also had significantly higher seed Mn (ranging from 57.9 mg kg^–1^ in *C. chorassanicum* to 162 mg kg^–1^ in *C. pinnatifidum)* than cultivated chickpea (37.1 mg kg^–1^). The wild species, except for *C. echinospermum* (3.1 mg kg^–1^) and *C. bijugum* (3.6 mg kg^–1^), also had significantly higher seed Cu, ranging from 4.3 mg kg^–1^ in *C. reticulatum* to 7.7 mg kg^–1^ in *C. judaicum*, than cultivated chickpea (3.4 mg kg^–1^). The eight wild Cicer species had significantly higher seed Ca, ranging from 3.02 g kg^–1^ in *C. echinospermum* to 6.09 g kg^–1^ in *C. chorassanicum*, than cultivated chickpea (2.22 g kg^−1^), and five wild Cicer species—*C. bijugum, C. chorassanicum, C. judaicum, C. pinnatifidum*, and *C. reticulatum*—had significantly higher seed Mg (1.65–1.83 g kg^–1^) than cultivated chickpea (1.41 g kg^–1^) (Sharma et al., 2021). The wide range of genetic variability in micronutrients found in various chickpea landraces and wild species presents opportunities for developing chickpea varieties to combat micronutrient-related ‘hidden hunger’.

## Vitamins

Chickpea is recognized as an important source of various vitamins, including niacin, riboflavin, folic acid, thiamin, and β-carotene (Cabrera et al., 2003; Abbo et al., 2005; Jha et al., 2015; Rezaei et al., 2016).

### β-carotene (precursor of vitamin A)

Chickpea contains significant amounts of β-carotene, the precursor of vitamin A, with reported wide ranges in carotenoid content from 22 μg g^–1^ (yellow cotyledon kabuli) to 44 μg g^–1^ (green cotyledon desi) (Rezaei et al., 2016). Lutein and zeaxanthin are the predominant components of carotenoids present in chickpea seeds, with green cotyledon chickpea cultivars exhibiting the highest provitamin A, including β-carotene and β-cryptoxanthin (Rezaei et al., 2016). Across five chickpea cultivars, CDC Jade had the highest lutein and β-cryptoxanthin in cotyledons at 16 days post-anthesis, while CDC Cory had the highest zeaxanthin and β-carotene (Rezaei et al., 2016). The same research group reported a wide range of total carotenoids in three segregating F_2_ populations (CDC Jade × CDC Frontier, CDC Cory × CDC Jade, and ICC4475 × CDC Jade), ranging from 10.6–40 µg g^–1^ in parental lines and 18.46–77.63 µg g^–1^ in segregating populations (Rezaei et al., 2019). Subsequently, Roorkiwal et al. (2022) identified wide genetic variation in β-carotene (0.003–0.104 mg 100 g^–1^) in 258 chickpea accessions using GWAS. Cooking chickpeas significantly reduces their vitamin A content from 67 IU 100 g^–1^ (uncooked) to 27 IU 100 g^–1^ (cooked) (Wallace et al., 2016).

### Vitamin B complex

Chickpea exhibits a wide range of genetic variation for various B vitamins, including thiamine (B1), riboflavin (B2), and niacin (B3). They are rich sources of thiamin (453 μg) and riboflavin (173 μg) (Hall et al., 2017). A recent GWAS assessing 258 chickpea accessions revealed significant variability for these vitamins, ranging from 0.055–0.502 mg 100 g^–1^ thiamine (average 0.189), 0.011–0.638 mg100 g^–1^ riboflavin (average 0.111), and 0.116–1.57 mg100 g^–1^ niacin [average 0.411 (Roorkiwal et al., 2022)]. Moreover, a comparative analysis of four chickpea genotypes revealed significant variability for thiamine, ranging from 0.31–0.36 mg 100 g^–1^ (Xiao et al., 2023). Moreover, one study reported significant genetic variation in various B vitamins between desi and kabuli types, with 1.72 and 1.22 mg 100 g^–1^ niacin, 0.21 and 0.26 mg 100 g^–1^ riboflavin, 0.29 and 0.49 mg 100 g^–1^ thiamin, and 0.30 and 0.38 mg 100 g^–1^ pyridoxine, respectively (Jukanti et al., 2012). However, cooking chickpeas reduces the levels of these vitamins significantly from 1.54 to 0.52 mg 100 g^–1^ niacin, 0.2 to 0.063 mg 100 g^–1^ riboflavin, and 1.58 to 0.28 mg 100 g^–1^ pantothenic acid (Wallace et al., 2016).

### Folic acid

Pulses are a significant source of dietary folate, with a wide range of folate levels documented across various pulse crops, including lentil (146–290 μg 100 g^–1^), yellow pea (50–202 μg 100 g^–1^), mung bean (141–169 μ 100 g^–1^), cowpea (96 μg 100 g^–1^), and common bean (103 μg 100 g^–1^) (Sen Gupta et al., 2013; Hefni and Witthoft, 2014). Folate content in chickpea ranges from 42–537 μg 100 g^–1^ (Hall et al., 2017), with cooking reducing the level from 557 μg 100 g^–1^ (uncooked) to 172 μg 100 g^–1^ (cooked) (Wallace et al., 2016). Moreover, chickpea consumers intake 627 µg day^–1^ folate compared to non-chickpea consumers (Wallace et al., 2016). Jha et al. (2015) used ultra-performance liquid chromatography and mass spectrometry to quantify six folate monoglutamates in four chickpea genotypes, revealing substantial genetic variability for folate (351–589 μg 100 g^–1^), with 5-methyltetrahydrofolate (5-MTHF) and 5-formyltetrahydrofolate (5-FTHF) the most abundant. Moreover, the study identified a significant effect of location and a location×cultivar interaction, suggesting that folate levels are responsive to genotype×environment interactions (Jha et al., 2015).

### Vitamin E

Chickpea oil is particularly rich in tocopherols, with alpha-tocopherol the highest among pulses, reaching up to 13.7 mg 100 g^–1^ (Wood and Grusak, 2007; Pittaway et al., 2008). The oil comprises four different forms of tocopherols—alpha, beta, gamma, and delta (Zia-Ul-Haq et al., 2009), with gamma-tocopherol recognized as a natural seed antioxidant (Gul and Egesel, 2008; Boschin and Arnoldi, 2011). The total tocopherol in chickpea oil ranges from 20.69–52.44 mg kg^–1^ (Gul and Egesel, 2008), with higher amounts of vitamin E in uncooked chickpea (0.82 mg 100 g^–1^) than cooked chickpea (0.35 mg 100 g^–1^) (Wallace et al., 2016). The National Health and Nutrition Examination Survey reported that chickpea consumers intake 10.1 μg day^–1^ more vitamin E than non-chickpea consumers (Wallace et al., 2016). The significant genetic variability observed for these vitamins underscores the potential of chickpea for addressing nutritional deficiencies, especially in regions where vitamin-related deficiencies are prevalent.

## Bioactive compounds

Chickpea is rich in bioactive compounds, including antioxidants, phenolic acids, flavonoids, and condensed tannins, which offer numerous health benefits (Heiras-Palazuelos et al., 2013). These compounds play significant roles in physiological and metabolic processes and contribute to reducing the risk of various diseases. Antioxidants in small quantities prevent the formation of free radicals or reactive oxygen species by retarding the oxidation of unsaturated fats, which are easily oxidized. Desi and kabuli chickpea exhibit varying antioxidant activities (Heiras-Palazuelos et al., 2013).

Phenols, essential bioactive compounds found in the chickpea seed coat (Xu et al., 2007), include phenolic acids, flavonoids, and condensed tannins (Singh et al., 2017; de Camargo et al., 2022), which offer various health benefits, including anti-carcinogenic, anti-thrombotic, anti-ulcer, anti-atherogenic, anti-allergenic, anti-inflammatory, antioxidant, and immune-modulating properties (de Camargo et al., 2022; Begum et al., 2023). Total phenolic contents in chickpea range from 27.48–48.01 mg 100 g^–1^ for kabuli types, 38.59–83.52 mg 100 g^–1^ for desi types, and 63.08–113.30 mg 100 g^–1^ for wild species (Kaur et al., 2019). They also observed that genotypes with higher phenol content exhibited greater antioxidant activity, suggesting a positive association between seed phenol content and antioxidant activity.A study using UPLC-MS/MS identified three specific phenolic acids in chickpea—taxifolin, biochanin, and m-hydroxybenzoic acid—that decrease oxidative damage in human HuH-7 cells induced by peroxy radicals, indicating hepatoprotective properties (de Camargo et al., 2022).

## Anti-nutrients

Chickpea seeds also contain substances like tannins, protease inhibitors (such as trypsin and amylase inhibitors), phytic acid, and saponins (Soetan and Oyewole, 2009; Kaur et al., 2014, Kaur et al, 2019) that function as anti-nutrients by inhibiting the bioavailability of various nutrient components (Soetan and Oyewole, 2009). Tannins are recognized as significant anti-nutritional compounds, binding with enzyme proteins or minerals, resulting in the inactivation of digestive enzymes and reduced protein digestion (Kaur et al., 2019). Various studies have reported tannin contents in chickpea seeds ranging from 5.44–10.87 mg g^–1^ (Kaur et al., 2014), 17.52 mg g^–1^ (Bulbula and Urga, 2018), and 11.93 mg g^–1^ in desi types, 10.63 mg g^–1^ in kabuli types, and 16.38 mg g^–1^ in wild species (Kaur et al., 2019). Chickpea genotypes also varied in tannin contents, ranging from 9.08 mg g^–1^ in GL 14015 to 18.47 mg g^–1^ in *C. judaicum* ILWC 30 (Kaur et al., 2019).

Phytic acid forms complexes with proteins, impeding the absorption of micronutrients such as Fe, Ca, Zn, Cu, and Mg in the gastrointestinal tract (Tiwari and Singh, 2012). Studies have reported phytic acid levels ranging from 3.49–11.52 mg g^–1^ in desi types and 3.45–12.35 mg g^–1^ in kabuli types (Mondor et al., 2009), 11.33 mg g^–1^ in whole chickpeas, 11.53 mg g^–1^ in split chickpeas, and 14 mg g^–1^ in desi types (Shi et al., 2018), and 9.43–13.67 mg g^–1^ in kabuli types, 8.48–18.39 mg g^–1^ in desi types, and 4.24–8.48 mg g^–1^ in wild species (Kaur et al., 2019). Phytic acid has a negative association with most minerals except Zn; an increase in phytic acid suggests a negative impact on the absorption of these minerals (Kaur et al., 2019).

Saponins, naturally occurring surface-active glycosides, also inhibit nutrient absorption and bioavailability in chickpea (Choudhary et al., 2015; Katoch et al, 2016; Kaur et al., 2019; Singh et al., 2022). They contribute to enzyme inactivation, significantly impacting cellular metabolism and reducing nutrient absorption. Studies have reported saponin levels ranging from 4.98–12.23 mg g^–1^ in kabuli types (Choudhary et al., 2015) and 7.22 mg g^–1^ in desi types, 7.02 mg g^–1^ in kabuli types, and 8.38–9.68 mg g^–1^ in wild species (Kaur et al., 2019).

Trypsin inhibitors hinder digestive enzymes, specifically trypsin and chymotrypsin, affecting the utilization of sulfur amino acids in the body. Genetic variability for trypsin inhibitors in chickpea ranges from 111.5–218.4 trypsin inhibitor units (TIU) g^–1^ (Gupta et al., 2017), 38.53–64.47 TIU g^–1^ in kabuli types, 32.91–112.32 TIU g^–1^ in desi types, and 122.73–150.18 TIU g^–1^ in wild species (Kaur et al., 2019). Thus, minimizing anti-nutrient contents through breeding, genomics, and other innovative approaches could improve the bioavailability of essential micronutrients, vitamins, phosphorus, and other nutrients, enhancing their nutritional values and health benefits (**Table 2**).

**Table 2:** Essential amino acid components in chickpea (g 100 g–1 protein).

Amino acids	Zia-Ul-Haq et al (2007)	Xiao et al (2023)	Grewal et al (2023)	Recommended amount (FAO, 2013)
Arginine	8.0–8.5	2.10–2.65	–	–
Histidine	2.9–3.2	0.65–0.79	2.89	2.0
Isoleucine	4.5–4.8	0.92–0.98	4.22	3.2
Leucine	8.1–8.5	1.45–1.70	7.64	6.6
Lysine	6.7–7.0	1.40–1.75	7.30	5.8
Methionine	0.8–1.1	0.23–0.37	Met + Cys (2.63)	2.7
Phenylalanine	5.0–5.3	1.20–1.50	Try + Phe (6.56)	5.2
Threonine	2.7–3.0	0.63–0.82	3.63	3.1
Tryptophan	0.8–0.9	0.57–0.81	1.41	0.85
Valine	4.1–4.6	0.99–1.10	3.80	4.3
Total	44.0–46.2	–	–	–

## Genomic resources for improving nutrients

Advancements in genome sequencing technologies have revolutionized chickpea breeding by providing extensive genomic resources, including decoding the chickpea genome sequence, whole genome resequencing, pan-genome assembly, and transcriptome assembly for various traits (Varshney et al., 2013; Varshney et al, 2021). Leveraging these advanced genomic resources has made it possible to dissect various traits of agronomic importance, including nutrient components in chickpea. For example, Wang et al. (2019) genotyped a bi-parental mapping population developed from ICC5912 × ICC995 across four environments, identifying several SPC QTL explaining 34.8–57% phenotypic variation explained (PVE) with the quantitative trait loci (QTL) *q-3.2* identified as the major seed protein QTL. Sab et al. (2020) identified 11 QTLs for seed Fe concentration on CaLG03, CaLG04, and CaLG05 using a F_2:3_ derived from MNK-1 and Annigeri 1, which explained 7.2% (*CaqFe3.4*) to 13.4% (*CaqFe4.2*) PVE. Further, eight QTLs for seed Zn, reported on CaLG04, CaLG05, and CaLG08, explained 5.7% (*CaqZn8.1*) to 13.7% PVE (*CaqZn4.3*) (Sab et al., 2020). Introgression of the *QTL-hotspot* region on CaLG04 that harbors several drought tolerance-related QTLs (Varshney et al., 2019) may increase seed Fe and Zn, with three QTLs for seed Fe and Zn (*CaqFe4.4, CaqFe4.5*, and *CaqZn4.1*) co-localized in this region (Sab et al., 2020). The authors identified genes in the QTL regions that encode Fe–S metabolism and Zn-dependent alcohol dehydrogenase activity on CaLG03, Fe ion binding oxidoreductase on CaLG04, and Zn-induced facilitator-like protein and ZIP zinc/iron transport family protein on CaLG05. Whole genome resequencing data identified 48 SNPs associated with targeted sugar types and nine genes “(*Ca_06204, Ca_04353*, and *Ca_20828*: *Phosphatidylinositol N-acetylglucosaminyltransferase*; *Ca_17399* and *Ca_22050*: *Remorin proteins*; *Ca_11152*: *Protein-serine/threonine phosphatase; Ca_10185, Ca_14209*, and *Ca_27229*: *UDP-glucose dehydrogenase*)” as potential candidates for sugar metabolism and transport in chickpea (Elango et al., 2022). A GWAS using 16,376 single nucleotide polymorphisms (SNP) markers identified seven genomic regions associated with SPC in 336 chickpea accessions, explaining a combined 41% PVE (Upadhyaya et al., 2016b) (see **Table 3**).

**Table 3:** List of QTL/genomic regions contributing to various nutritional components in chickpea.

Nutrient component	Population type	QTL/genomic region	LG/Chromosome and name and position of associated marker	PVE%	References
Protein content	GWAS (187)	Two significant MTAs	LG3, 5	8–17	Jadhav et al. (2015)
Protein content	GWAS (336)	Seven genomic regions	Chromosomes 1, 2, 4, 6, 7	41	Upadhyaya et al. (2016b)
Protein content	ICC 995×ICC5912, RIL (189)	*qPRO-ABE-1.1, qPRO-ABE-3.2, qPRO-BIG-3.2, qPRO-BIG_6.2, qPRO-GH-1.1, qPRO-GH-3.2, qPRO-ICR-1.1, qPRO-ICR-3.2, qPRO-1.1, qPRO-3.1, qPRO-3.2, qPRO-6.2*	LG1, 3, 6	34.8–57	Wang et al. (2019)
			LG1 (Ca1:363821–Ca1:1162304, Ca1:363821–Ca1:1162304, Ca1:363821–Ca1:1162304)		
			LG3 (Ca3:22724462–CaGM13632, Ca3:22724462–CaGM13632, Ca3:22724462–CaGM13632, Ca3:22724462–CaGM13632, Ca3:7351552–Ca3:10193673, Ca3:22724462–CaGM13632)		
			LG6 (Ca6:2432945–Ca6:3140044)		
Protein content	GWAS	7 SNPs	Chromosomes 1, 4, 6, 7		Srungarapu et al (2022b)
Protein content	ICC4958 × ICC12299, RIL (180) GWAS (211)	*CaREN1*	LG5 (SNP1 genomic location 37177950, SNP5, 195644) LG6 (SNP1 genomic location 37177950, SNP2, 11095309, SNP3, 11106054)	23	Chakraborty et al. (2023)
Protein content (under heat stress)	GWAS (140)	15 MTAs	Chromosomes 1, 2, 3, 6, 7 S3_10482045 and S6_59061568	24	Samineni et al. (2022)
Protein content (under drought stress)	GWAS (140)	46 MTAs	Chromosomes 6, 7	18	Samineni et al. (2022)
			S1_1451316, S1_18239723, S1_812178, S1_35622241, nd S6_ 12788060		
Protein content (under non stress)	GWAS (140)	66 MTAs	Chromosome 3 (S3_28729262, S3_5346023; S3_5969219) Chromosome 4 (S4_38618901) Chromosome 5 (S5_3793636) Chromosome 6 (S6_46910162) Chromosome 8 (S8_13109034)	1–28	Samineni et al. (2022)
Protein content	GWAS (258)	4 MTAs	Ca6 (Ca6_57802709)	1.7–2.4	Roorkiwal et al. (2022)
Protein content	GWAS (88)	3 MTAs	LG1, 4, 6 SCA1_V1.0_KABULI_27758317 SCA4_V1.0_KABULI_10255941 SCA6_V1.0_KABULI_55623192	10.6–11.5	Mugabe et al. (2023)
Starch content	ICC995×ICC5912, RIL (189)	*q-1.1*	Chromosome 1 (markerCa1:333974)	14.3–31.6	Wang et al. (2019)
Raffinose	GWAS (211)	16 SNPs changes in 31 genes	SNP Ca4_43438450	–	Elango et al. (2022)
Stachyose	GWAS (211)	7 SNPs changes in 12 genes	–	–	Elango et al. (2022)
Sucrose	GWAS (211)	9 SNPs changes in 17 genes	SNP Ca6_2510863	–	Elango et al. (2022)
Total sugars	GWAS (211)	8 SNPs changes in 14 genes	–	–	Elango et al. (2022)
Starch content	GWAS (88)	MTAs	Chromosomes 1, 4, 5, 6 SCA1_V1.0_KABULI_27758317 SCA4_V1.0_KABULI_38788991 SCA5_V1.0_KABULI_28817538 SCA6_V1.0_KABULI_4964048	4.6–19.7	Mugabe et al. (2023)
Oil content	GWAS (88)		LG2, 5 SCA2_V1.0_KABULI_17862582 SCA2_V1.0_KABULI_6644653 SCA5_V1.0_KABULI_4127731	7.6–16	Mugabe et al. (2023)
Fatty acid (palmitic acid, linoleic acid, alpha-linolenic acid)	GWAS (354)	5 significant SNPs	Chromosomes 1, 2, 8 Chromosome 2 (SCM001765.1_7756123, SCM001765.1_7281701) Chromosome 8 (SCM001771.1_3705203, SCM001771.1_13092034) Chromosome 1 (SCM001764.1_29706924)		Salaria et al. (2023)
Calcium content	GWAS (258)	One MTA	Ca5	1.6	Roorkiwal et al. (2022)
Copper content	GWAS (147)	12 MTAs	LG1, 2, 3, 4, 5, 6, 8 LG1 (Affx_123261919) LG2 (Affx_123255840) LG6 (Affx_123294330, Affx_123280871)	12.8–19.6	Fayaz et al. (2022)
Zinc and iron content	GWAS (96)	8 significant SNPs associated with Zn and Fe content	Chromosomes 1, 4, 6, 7	–	Diapari et al. (2014)
Zinc and iron content	GWAS (92)	16 genomic loci/genes	LG1, 2, 3, 4, 5, 7	29	Upadhyaya et al. (2016a)
Zinc and iron content	ICC 4958 × ICC 8261 RIL (277)	*CaqFe1.1*, *CaqZn2*.1, *CaqFe3*.1, *CaqZn3.1, CaqFZ4.1. CaqFe4.1, CaqFZ5.1, CaqFZ7.1*	LG1, 2, 3, 4, 5, 7	16.9–23.6	Upadhyaya et al. (2016a)
Zinc content	GWAS (107 C*. reticulatum* and 73 C*. arietinum*)	23 significant SNPs related to seed Fe content	Chromosomes 1, 4, 5 Chromosome 1 (SNP204) Chromosome4 (SNP8254, SNP8255) Chromosome 5 (SNP 9478)	7.3–21	Karaca et al. (2020)
Zinc content		16 significant SNPs related to seed Zn content	Chromosomes 2, 3, 4 Chromosome 4 (SNP8284) Chromosome 5 (SNP9528, SNP9529, SNP10249)	7–16	Karaca et al. (2020)
Zinc content	GWAS (147)	5 MTAs	LG1, 4, 7 LG4 (Affx_123261732) LG7 (Affx_123247267, Affx_123295749, Affx_123241958) LG1 (Affx_123243695)	11.6	Fayaz et al. (2022)
Iron content	GWAS (147)	9 MTAs	LG1, 3, 4, 5, 6, 8 LG5 (Affx_123275255) LG6 (Affx_123293935, Affx_123243960, Affx_123240923) LG4 (Affx_123282040) LG3 (Affx_123258734) LG1 (Affx_123272321) LG8 (Affx_123255008)	up to 20	Fayaz et al. (2022)
Manganese content	GWAS (147)	9 MTAs	LG1, 2, 3, 4, 7 LG7 (Affx_123292401, Affx_123261947) LG3 (Affx_123296790, Affx_123293942) LG4 (Affx_123291734, Affx_123252620, Affx_123272607) LG2 (Affx_123296837) LG1 (Affx_123282242)	4.5–12.7	Fayaz et al. (2022)
Zinc content	GWAS (258)	7 MTAs	Ca1, 3, 6 (Ca1_1204130, Ca3_31771545)	2.6–12.1	Roorkiwal et al. (2022)
Mn content	GWAS (258)	–	Ca2_7953148		Roorkiwal et al. (2022)
Iron content	GWAS (258)	1 MTA	Ca1	–	Roorkiwal et al. (2022)
Iron content (under heat stress)	GWAS (140)	43 MTAs	S1_12185432, S3_37090540	22	Samineni et al. (2022)
Zinc content (under heat stress)	GWAS (140)	5 significant SNPs	Chromosome 2 (S2_2323804, S2_2370534, S2_2312104) Chromosome 4 (S4_32672776) Chromosome 7 (S7_37159003)	–	Samineni et al. (2022)
Iron content (under drought stress)	GWAS (140)	1 MTA	Chromosome 4	9	Samineni et al. (2022)
Zinc content (under drought stress)	GWAS (140)	1 MTA	Chromosome 7	11	Samineni et al. (2022)
Iron content (under non stress)	GWAS (140)	1 MTA	Chromosome 4 (S4_44607232)	11	Samineni et al. (2022)
Zinc content (under non stress)	GWAS (140)	3 MTAs	Chromosomes 1, 7, 4 (S1_15267578, S7_11907729, S4_9867593)	34	Samineni et al. (2022)
Iron content	GWAS	12 SNPs	Chromosomes 1, 4, 6, 7	–	Srungarapu et al (2022b)
Zinc content	GWAS	1 SNP	–	–	Srungarapu et al (2022b)
Folate content	GWAS (258)	10 MTAs	Ca2, 4, 5, 6 (Ca5_664616, Ca4_1677219)	3.5–28.6	Roorkiwal et al (2022)
Vitamin B2	GWAS (258)	10 MTAs	Ca1, 3, 4, 6 (Ca3_3519666)	up to 25	Roorkiwal et al (2022)
Vitamin B6	GWAS (258)	14 MTAs	Ca1, 3, 4, 5, 7 (Ca4_17620596)	up to 9.5	Roorkiwal et al. (2022)
Vitamin B1	GWAS (258)	4 MTAs	Ca1, 2, 4 (Ca1_32272158)	–	Roorkiwal et al. (2022)
Manganese	GWAS (258)	4 MTAs	Ca1, 2, 4, 6	11.4	Roorkiwal et al. (2022)
Magnesium	GWAS (258)	2 MTAs	Ca4	8.5	Roorkiwal et al. (2022)
Beta carotene	*Cicer arietinum* L.×	4 QTLs for beta-carotene	LG1b., LG3	–	Abbo et al. (2005)
Lutein	*C. reticulatum*	1 QTL for lutein concentration	LG8	–	Abbo et al. (2005)
Carotenoid content	Biparental mapping populations CDC Jade’ × ‘CDC Frontier’ CDC Cory’ × ‘CDC Jade’ ‘ICC4475’ × ‘CDC Jade	8 QTLs (β-carotene, zeaxanthin, β-cryptoxanthin, β-carotene, violaxanthin)	LG1,3,5 and 8 LG1 (marker AX-123644659 for *q-Zea-1-JF* QTL, AX-123641029 for q-Crt-1-JF QTL, AX-123638575 for q-Lut-1-JF QTL) LG5 (marker AX-123632228 for q-Zea-5-JF QTL) LG8 (marker AX-123637790 for q-Cryp-8-JF QTL, AX-123657409 for q-Crt-8-JF QTL, AX-123657409 for q-Vio-8-JF QTL)	58–70	Rezaei et al. (2019)
β-carotene	GWAS (258)	2 MTAs	Ca4, 5	2.4	Roorkiwal et al. (2022)
Phytic acid	GWAS (258)	1 MTA	Ca1	–	Roorkiwal et al. (2022)
Fiber content	GWAS (88)	MTAs	Chromosome 7 (SCA7_V1.0_KABULI_33227889, SCA7_V1.0_KABULI_39055774)	20	Mugabe et al. (2023)
				8.5–19.8	
Ciceritol	GWAS (211)	7 SNPs changes in 12 genes	SNP Ca_46225454, Ca5_1870839	–	Elango et al. (2022)

Furthermore, a combined QTL-seq and GWAS approach identified two major QTLs that regulate SPC on LG5 and LG6 (Chakraborty et al., 2023). The highly significant SNPs and genomic region controlling SPC were validated in 211 chickpea accessions, indicating the tight association of an SNP marker with the “*CaREN1* (*ROP1 ENHANCER1*)” genomic region, explaining 23% PVE. *CaREN1* knockdown significantly reduced SPC, confirming the role of *CaREN1* in governing SPC (Chakraborty et al., 2023).

Diapari et al. (2014) conducted GWAS using SNP markers to elucidate genomic regions associated with grain Zn and Fe in 96 chickpea genotypes, identifying eight SNPs contributing to Zn or Fe, with one SNP on chromosome 1 associated with both. Additionally, two SNPs related to Fe and Zn were identified on chromosomes 6 and 7, and three SNPs related to Zn and two SNPs related to Fe were identified on chromosome 4 (Diapari et al., 2014) (see **Table 3**).

A recombinant inbred lines-based bi-parental mapping population approach using an ICC 4958 × ICC 8261 population identified eight QTLs governing seed Fe and Zn on six chromosomes, explaining a combined 39.4% PVE (Upadhyaya et al., 2016a). Furthermore, genotyping 92 sequenced desi and kabuli accessions with 24,620 SNPs identified 16 genomic loci/genes contributing to seed Fe and Zn, accounting for a combined 29% PVE (Upadhyaya et al., 2016a). Subsequently, a GWAS on a diverse panel of 147 chickpea genotypes phenotyped for two years and genotyped with an “Axiom^®^50K CicerSNP array” identified 35 significant marker-trait associations (MTAs) contributing to grain Zn, Fe, Cu, and Mn, with five MTAs consistently identified in different environments (stable), six explaining more than 15% of the phenotypic variation (major), and three both stable and major MTAs (Fayaz et al., 2022). Likewise, over two years, SNP204 on LG1 and SNP9478 on LG5 showed significant MTAs for Fe content at Sanliurfa, and SNP8254 and SNP8255 on LG4 showed significant MTAs for Fe content at Bornova (Karaca et al., 2020). A GWAS on 258 chickpea genotypes using 318,644 SNPs derived from whole genome sequencing revealed 62 significant MTAs for 12 important nutritional traits, including crude protein, β-carotene, seed Ca, and folate content, on chromosomes Ca1, Ca3, Ca4, and Ca6, explaining up to 29% PVE (Roorkiwal et al., 2022). A GWAS on a reference set of 280 chickpea genotypes using a 5k SNP array and the FarmCPU and BLINK models identified seven significant SNPs for grain protein, 12 SNPs for Fe, and one SNP for Zn on chromosomes 1, 4, 6, and 7 (Srungarapu et al., 2022b). Another GWAS analysis identified 181 MTAs for grain protein, Zn, and Fe content in 140 diverse chickpea genotypes under non-stress, drought, and heat stress conditions, with 48 and 63 MTAs significantly associated with drought stress and heat stress, respectively (Samineni et al., 2022). Thus, targeting the identified overlapping/common genomic regions controlling these micronutrients for cloning could help elucidate the precise functions of candidate genes associated with nutrient content.

A GWAS on 354 kabuli and desi chickpea genotypes highlighted the significance of key grain fatty acids, including palmitic, linoleic, alpha-linolenic, and oleic acids, identifying five significant SNPs on chromosomes 1, 2, and 8 strongly associated with palmitic acid (Salaria et al., 2023). Another GWAS using 36,645 SNP markers derived from 88 chickpea accessions uncovered MTAs for starch on chromosome 2 (12% PVE), fiber content on chromosome 6 (20% PVE), oil content on chromosome 7 (11% PVE), and grain protein on chromosome 1 (11% PVE) (Mugabe et al., 2023).

## Functional genomics approaches for discovering candidate gene(s) contributing to nutrients

Advances in functional genomics, particularly RNA-seq-based transcriptome assembly, have greatly enhanced our ability to identify trait-based candidate genes in chickpea (Kudapa et al., 2018). For instance, quantitative RT-PCR can be used to profile the expression of candidate genes for SPC. Notably, GWAS revealed higher differential upregulatory expression in high SPC-containing mapping individuals (21.5–22.4%) than in low SPC-containing mapping individuals (15.6–16.5%) during the seed development stage (Upadhyaya et al., 2016b). Similarly, functional analysis of the SPC gene *ROP1ENHANCER1*, identified through combined QTL-seq and candidate gene-based association mapping, demonstrated a significant reduction in SPC when this gene was knocked down in chickpea (Chakraborty et al., 2023). Similarly, Upadhyaya et al. (2016a) used qRT-PCR to explore the functional expression of candidate genes related to grain Fe and Zn, reporting high expression in the seeds of parental chickpea genotypes with high Fe and Zn compared to those with low Fe and Zn.

Furthermore, investigating the potential role of various transporter genes (“*FRO2, IRT1, NRAMP3, V1T1, YSL1, FER3, GCN2*, and *WEE1”*) in Fe metabolism, Jahan et al. (2023) validated their function in Fe uptake, root and stem translocation, and leaf tissue accumulation. Examining the expression patterns of identified genes related to carotenoid content, Rezaei et al. (2016) analyzed the expression of 19 selected genes associated with the carotenoid biosynthesis pathway in five chickpea cultivars, reporting up-regulatory expression in the CDC Jade cultivar.

Functional genomics advancements will potentially uncover more candidate genes associated with quality traits, offering insights into their precise functions. This knowledge could facilitate the cloning and transfer of these genes to elite chickpea cultivars.

## Innovative breeding tools for improving nutritional components

Recent advances in breeding approaches, including genomic selection, speed breeding, and high-throughput phenotyping, offer promising avenues for improving the nutritional components of chickpea and developing nutritionally dense or biofortified genotypes. Genomic selection (GS) can harness high-throughput SNP markers derived from chickpea genomics resources to select progenies with superior genetic merit for various nutritional traits using prediction models trained on a large target population (Meuwissen et al., 2001). Speed breeding protocols can expedite the generation of mapping populations, such as recombinant lines and backcross populations, for mapping various nutritional component QTLs/genes (Watson et al., 2018). Advances in high-throughput phenotyping and non-destructive phenotyping, including hyperspectral imaging, Fourier transform near-infrared imaging, and micro-computed tomography imaging, offer efficient means of assessing nutritional components in chickpea (Hacisalihoglu and Armstrong, 2023). Emerging approaches like artificial intelligence and machine learning tools that use convolutional and deep neural networks could predict nutritional quality and the role of novel genes/pathways associated with various nutritional and anti-nutritional components in chickpea (Tachie et al., 2023). By integrating these innovative breeding tools into chickpea breeding programs, researchers can accelerate the development of nutritionally enhanced varieties, contributing to efforts to combat hunger and improve food security worldwide.

## Scope of genome editing for improving nutrient bioavailability

Conventional breeding approaches have significantly increased the global yield and production of chickpea. Before the advent of CRISPR/Cas9 (Wang et al., 2016), other genome editing systems like zinc-finger nucleases (ZFNs) (Urnov et al., 2010), transcription activator-like effector nucleases (TALENs) (Joung and Sander, 2013), and homing endonucleases or meganucleases (Pâques and Duchateau, 2007; Daboussi et al., 2015) were used. Zinc-finger nucleases, comprising distinct DNA binding and *FokI* nuclease DNA cleavage domains, were among the earliest synthetic proteins used for targeted mutagenesis and gene replacement (Li et al., 1992; Carroll, 2011; Davies et al., 2017). However, they suffered from low specificity, limited efficacy, and inability to achieve gene knockout and RNA editing (Wang et al., 2016; Salsman and Dellaire, 2017; Kumar et al., 2022). Similarly, TALENs, which function as nonspecific DNA-cleaving nucleases, tether a restriction nuclease to a DNA-binding protein domain termed TAL effector (Hensel and Kumlehn, 2019) but faced similar drawbacks as ZFNs. Meganucleases, rare cutting enzymes also known as homing endonucleases (Pingoud and Silva, 2007), offered highly specific site cleavage (Khan, 2019) with low cytotoxicity. Despite their efficiency in excising large DNA sequences, challenges in manufacturing and potential off-targeting effects hindered their widespread use (Jin et al., 2016; Kumar et al., 2022).

In contrast, CRISPR/Cas9-based genome editing technology has revolutionized molecular biology by enabling precise and accurate targeted mutations in desired genomic regions (Wada et al., 2020). This breakthrough offers opportunities to improve functional quality traits, including nutrient-related parameters (Haun et al., 2014; Waltz, 2016; Sun et al., 2017; Li et al., 2018; Sánchez-León et al., 2018). Notably, a CRISPR/Cas9 protocol has been successfully established in chickpea (Badhan et al., 2021; Gupta et al., 2023a) and used to edit genes like *4-coumarate ligase* (*4CL*) and *Reveille 7* (*RVE7*) associated with drought tolerance (Badhan et al., 2021). Likewise, this technology has successfully knocked out chickpea phytoene desaturase (*CaPDS*), resulting in albino chickpea phenotypes (Gupta et al., 2023b).

Recent advances in genome editing, such as base editing and prime editing, further enhance the efficiency and precision of CRISPR/Cas9. Base editing introduces single nucleotide variants into DNA/RNA through programmable base editors (Porto et al., 2020), while prime editing enables insertions, deletions, or base conversions of up to 12 nucleotides without introducing double-strand breaks (Anzalone et al., 2019; Zong et al., 2022; Ahmad et al., 2023; Zhao et al., 2023). These technologies hold promise for addressing challenges in crop improvement, including developing biofortified chickpea varieties to combat malnutrition and promote global nutritional security. However, overcoming technical hurdles like reliable transformation and regeneration protocols, identifying genomic regions for target traits, and determining the precise metabolic pathways involved (Ku and Ha, 2020) remains crucial for realizing the full potential of genome editing in crop enhancement.

## Conclusions and future perspectives

Addressing the challenges of global food security and malnutrition requires concerted efforts to enhance the nutritional quality of crops like chickpea. While progress has been made in increasing chickpea yield, there remains a need to balance this with improved nutritional traits. Thorough studies on the correlation of various quality traits, including carbohydrates, proteins, fats, and micronutrients, are crucial for integrating these factors into efforts aimed at enhancing nutritional characteristics. Recognizing the tradeoff between production and quality traits is crucial, and breeding programs should aim to find the optimal balance to meet both needs.

**Figure 2:**
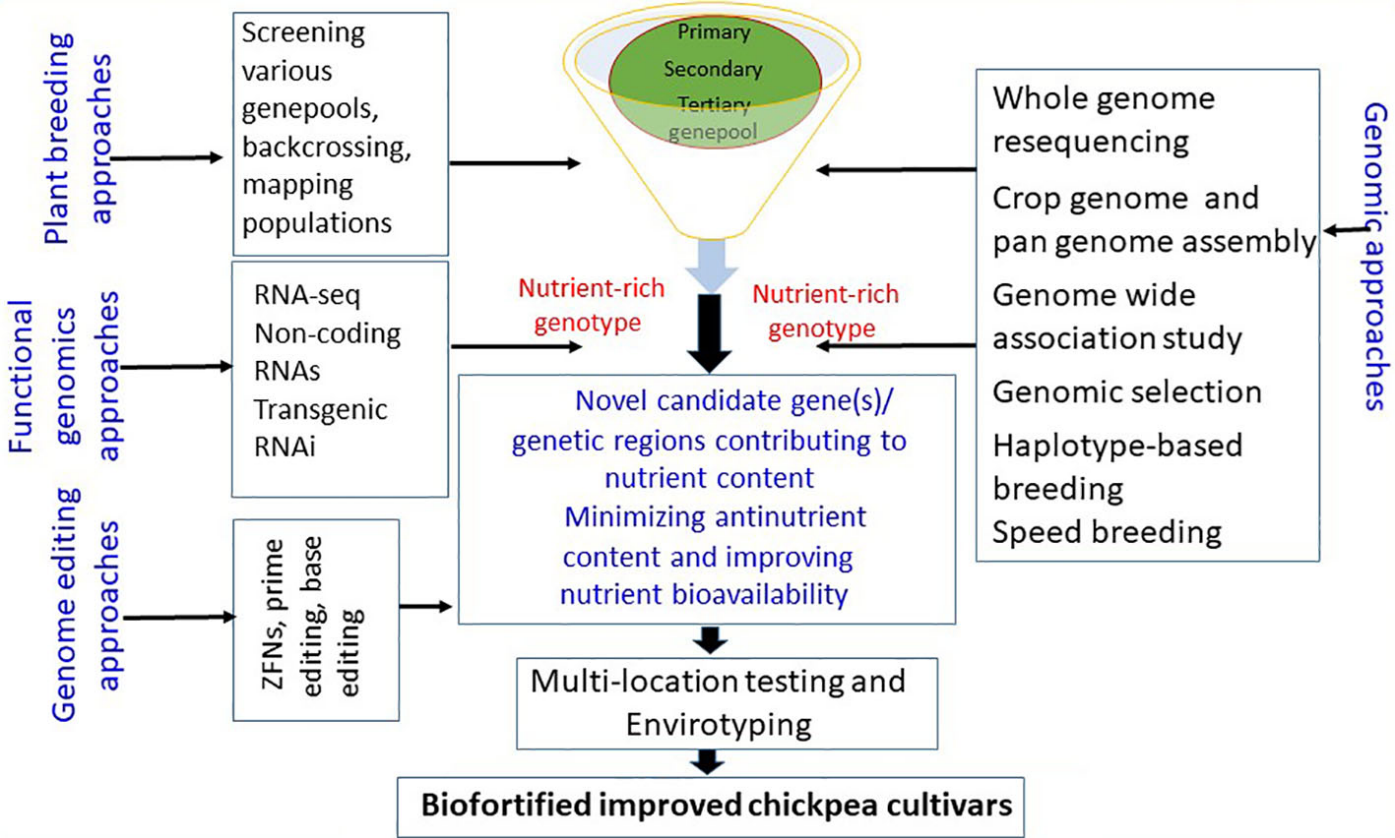
Various breeding and innovative approaches for developing biofortified chickpea cultivars.

Using the genetic diversity in chickpea crop wild relatives, landraces, and germplasm resources and leveraging genomic resources such as the chickpea genome sequence and pan-genome assembly can help identify the key genetic determinants/gene(s)/QTL controlling nutritional traits. Marker-assisted selection can facilitate the transfer of nutrient-dense genomic regions to elite chickpea cultivars (see **Figure 2**). Furthermore, CRISPR/Cas9 genome editing offers precise editing of genomic regions related to anti-nutrients, enhancing nutrient bioavailability. Efforts to elucidate metabolic pathways associated with quality traits will deepen our understanding of the molecular mechanisms and gene(s) governing these quality traits. Developing improved chickpea cultivars with enhanced nutrition can help meet the rising demand for protein-rich diets and combat malnutrition, contributing to global food and nutrition security, modern cropping system diversity, and agricultural sustainability.

## Author contributions

UJ: Writing – original draft, Writing – review & editing. HN: Writing – review & editing. MT: Writing – review & editing. BR: Writing – review & editing. PP: Writing – original draft, Writing – review & editing. KS: Writing – original draft, Writing – review & editing.
