# Supplementary material for: Unlocking the nutritional potential of chickpea: strategies for biofortification and enhanced multinutrient quality
Source: Front Plant Sci. 2024 Jun 7;15:1391496. doi: 10.3389/fpls.2024.1391496 (PMC11190093; doi:10.3389/fpls.2024.1391496)
Supplement: Supplementary file 1 [file Table_1.docx]

| **Supplementary Table. 1. List of various nutrient components in chickpea** | |  |
| --- | --- | --- |
| **Nutrient component** | **Amount/range** | **Reference** |
| Protein | 16.8–26.8 % | Ocampo et al. (1998) |
|  | 17.55–23.31% | Özer et al. (2010) |
|  | 15.6–22.4% | Upadhyaya et al. (2016b) |
|  | 4.60–33.90% | Bhagyawant et al. (2018) |
|  | 23.19–28% (desi type), 23.15–25.97% (kabuli type), 23.03–26.82% (wild species) | Kaur et al. (2019) |
|  | 21–25.5% (wild species) | Sharma et al. (2021) |
|  | 19.79–23.38 % | Xiao et al. (2023) |
|  | 16.56–24.64% | Roorkiwal et al. (2022) |
|  | 16.3–26.2% | Srungarapu et al. (2022a) |
|  | 11.6–24.8, 15.7–26.2, 15.9–24.7% | Samineni et al. (2022) |
|  | 18.9–32.4% | Farida Traoré et al. (2022) |
| Carbohydrates | 62 % | Patane (2006) |
|  | 31.80% and 35.24% total amylose and 29.93% and 31.11% apparent amylose for kabuli and desi, respectively | Miao et al. (2009) |
|  | Starch (41.76–49.07%) | Özer et al. (2010) |
|  | Starch (27.15–36.22%) | Xiao et al. (2023) |
|  | Sucrose (3.57–54.12 mg g^-1^)  Sucrose (0.6-3.59 g 100g^-1^) | Elango et al. (2022)  Gangola et al. (2013) |
|  | Raffinose (0.16–15.13 mg g^-1^ ) | Elango et al. (2022) |
|  | Stachyose (2.77–59.43 mg g^-1^)  Stachyose (0.18–2.38 g 100g^-1^ ) | Elango et al. (2022)  Gangola et al.(2013) |
| Fat/fatty acids | Palmitic (18.9–20.4%) | Zia-Ul-Haq et al. (2007) |
|  | Oleic (21.6–22.2%) | Zia-Ul-Haq et al. (2007) |
|  | Linolenic (0.5–0.9%) | Zia-Ul-Haq et al. (2007) |
|  | Linoleic (54.7–56.2%) | Zia-Ul-Haq et al. (2007) |
|  | Stearic (1.3–1.7%) | Zia-Ul-Haq et al. (2007) |
|  | Oleic (18.57% and 35.23%) | Gül et al. (2008) |
|  | Linoleic acid (47.15% and 63.44%) | Gül et al. (2008) |
|  | 4.45–6.11% | Özer et al. (2010) |
|  | 6.35–9.35 % | Xiao et al. (2023) |
|  | Oleic (24.81–35.98%) | Xiao et al. (2023) |
|  | Linoleic (49.3–58.18%) | Xiao et al. (2023) |
|  | Palmitic (10.35–12.23%) | Xiao et al. (2023) |
|  | Arachidic (0.78–0.97%) | Xiao et al. (2023) |
|  | Stearic (1.58–2.19%) | Xiao et al. (2023) |
|  | Palmitic (450.7–912.6 mg 100 g^-1^) | Salaria et al. (2023) |
|  | Linoleic (1605.7–3459.9 mg 100 g^-1^ ) | Salaria et al. (2023) |
|  | Alpha–linolenic (416.4–864.5 mg 100 g^-1^) | Salaria et al. (2023) |
|  | Oleic (1035.5–1907.2 mg 100 g^-1^) | Salaria et al. (2023) |
| Fiber | 2.88 % | Patane (2006) |
|  | 2.03–4.18% | Özer et al. (2010) |
| Ash | 2.64 % | Patane (2006) |
|  | 2.54–3.41% | Özer et al. (2010) |
|  | 2.59–2.69 % | Xiao et al. (2023) |
| Calcium | 109.6 mg 100 g^-1^ | Patane (2006) |
|  | 1.4– 3.4 mg g^1^ | Ereifej et al. (2001) |
|  | 185–219 mg 100 g^-1^ | Zia-Ul-Haq et al. (2007) |
|  | 93.4–197.4 mg 100 g^-1^ | Thavarajah and Thavarajah (2012) |
|  | 160.58 mg 100 g^-1^ | Marioli Nobile et al. (2013) |
|  | 40.83–260.95 mg 100 g^-1^ | Kaur et al. (2019) |
|  | 1.62–3.16 mg kg^-1^ (cultivated chickpea) | Sharma et al. (2021) |
|  | 3.54–4.63 mg kg^-1^ (*C. bijugum*) | Sharma et al. (2021) |
|  | 6.06–6.12 mg kg^-1^ (*C .chorassanicum*) | Sharma et al. (2021) |
|  | 3.19–3.58 mg kg^-1^ (*C. cuneatum*) | Sharma et al. (2021) |
|  | 2.37–3.58 mg kg^-1^ (*C. echinospermum*) | Sharma et al. (2021) |
|  | 4.52–5.96 mg kg^-1^ (*C. judaicum*) | Sharma et al. (2021) |
|  | 3.94–4.48 mg kg^-1^ (*C. pinnatifidum*) | Sharma et al. (2021) |
|  | 2.56–3.38 mg kg^-1^ (*C. reticulatum*) | Sharma et al. (2021) |
|  | 4.95–5.11 mg kg^-1^ (*C. yamashitae*) | Sharma et al. (2021) |
|  | 60.7–176.5 mg 100 g^-1^ | Roorkiwal et al. (2022) |
|  | 60.69–176.55  mg 100 g^-1^ | Roorkiwal et al. (2022) |
|  | 45.6 mg 100 g^-1^ | Devi et al. (2023) |
| Iron | 4.50 mg 100 g^-1^ | Patane (2006) |
|  | 42–141 μg g^1^ | Ereifej et al. (2001) |
|  | 2.4–4.1 mg 100 g^-1^ | Zia-Ul-Haq et al. (2007) |
|  | 4.6–6.7 mg 100 g^-1^ | Thavarajah and Thavarajah (2012) |
|  | 3.0–14.3 mg 100 g^-1^ | Diapari et al. (2014) |
|  | 48.6–56.6 mg kg^-1^ | Ray et al. (2014) |
|  | 4.56–9.87 mg 100 g^-1^ | Kaur et al. (2019) |
|  | 0.50–8.54  mg 100 g^-1^ | Grewal et al. (2020) |
|  | 33.3–46.7 mg kg^-1^ (cultivated chickpea) | Sharma et al. (2021) |
|  | 70.8–82.1 mg kg^-1^ (*C. bijugum*) | Sharma et al. (2021) |
|  | 56.7–78.3 mg kg^-1^ (*C.chorassanicum*) | Sharma et al. (2021) |
|  | mg kg^-1^ *cuneatum*) | Sharma et al. (2021) |
|  | 43–54.5 mg kg^-1^ (*C. echinospermum*) | Sharma et al. (2021) |
|  | 161.2–232.7 mg kg^-1^ (*C. judaicum*) | Sharma et al. (2021) |
|  | 65.8–82.8 mg kg^-1^ (*C. pinnatifidum*) | Sharma et al. (2021) |
|  | 46.8–63.6 mg kg^-1^ (*C. reticulatum*) | Sharma et al. (2021) |
|  | 160–171.8 mg kg^-1^ (*C. yamashitae*) | Sharma et al. (2021) |
|  | 14.61 mg 100 g^-1^ | Fayaz et al. (2022) |
|  | 2.26–7.25 mg 100 g^-1^ | Roorkiwal et al. (2022) |
|  | 44.1–76.7 mg kg^-1^ | Srungarapu et al. (2022b) |
|  | 47.8–83.0 (non-stress), 49.4–86.2 (drought stress), 41.4–77.6 (heat stress) mg kg^-1^ | Samineni et al. (2022) |
|  | 5.49–9.72 mg 100 g^-1^ | Xiao et al. (2023) |
|  | 3.16 mg 100 g^-1^ | Devi et al. (2023) |
| Zinc | 29.8–60.3 μg g^1^ | Ereifej et al. (2001) |
|  | 3.7–7.4 mg 100 g^-1^ | Thavarajah and Thavarajah (2012) |
|  | 21.1–28.3 mg kg^1^ | Ray et al. (2014) |
|  | 0.96–4.05 mg 100 g^-1^ | Kaur et al. (2019) |
|  | 1.10 to 5.91  mg 100 g^-1^ | Grewal et al. (2020) |
|  | 21.8–34.6 mg kg^-1^ (cultivated chickpea) | Sharma et al. (2021) |
|  | 29.6–30.1 mg kg^-1^ (*C. bijugum*) | Sharma et al. (2021) |
|  | 37.3–37.6 mg kg^-1^ (*C.chorassanicum*) | Sharma et al. (2021) |
|  | 29.6–32.3 mg kg^-1^ (*C. cuneatum*) | Sharma et al. (2021) |
|  | 30–37.6 mg kg^-1^ (*C. echinospermum*) | Sharma et al. (2021) |
|  | 20.6–30.6 mg kg^-1^ (*C. judaicum*) | Sharma et al. (2021) |
|  | 32.4–39.4 mg kg^-1^ (*C. pinnatifidum*) | Sharma et al. (2021) |
|  | 27.6–41 mg kg^-1^ (*C. reticulatum*) | Sharma et al. (2021) |
|  | 44.2–49.7 mg kg^-1^ (*C. yamashitae*) | Sharma et al. (2021) |
|  | 4.59 mg 100 g^-1^ | Fayaz et al. (2022) |
|  | 1.15–4.59 mg 100 g^-1^ | Roorkiwal et al. (2022) |
|  | 29.5–55.0, 28.1–63.1, 29.7–55.4 mg kg^-1^ | Samineni et al. (2022) |
|  | 36.3–56.2 mg kg^-1^ | Srungarapu et al. (2022b) |
|  | 3.61–5.33 mg 100 g^-1^ | Xiao et al. (2023) |
| Copper | 13.6– 26.6 μg g^1^ | Ereifej et al. (2007) |
|  | 10.7–12.2 mg 100 g^-1^ | Zia-Ul-Haq et al. (2001) |
|  | 0.7–1.1 mg 100 g^-1^ | Thavarajah and Thavarajah (2012) |
|  | 6.6–8.7 g kg^1^ | Ray et al. (2014) |
|  | 0.13–1.07 mg 100 g^-1^ | Kaur et al. (2019) |
|  | 2.6–4.9 mg kg^-1^ (cultivated chickpea) | Sharma et al. (2021) |
|  | 3.2–4 mg kg^-1^ (*C. bijugum*) | Sharma et al. (2021) |
|  | 6.1–6.6 mg kg^-1^ (*C. chorassanicum*) | Sharma et al. (2021) |
|  | 4.7–5.8 mg kg^-1^ (*C. cuneatum*) | Sharma et al. (2021) |
|  | 2.7–3.4 mg kg^-1^ (*C. echinospermum*) | Sharma et al. (2021) |
|  | 5.6–8.9 mg kg^-1^ (*C. judaicum*) | Sharma et al. (2021) |
|  | 5.1–6 mg kg^-1^ (*C. pinnatifidum*) | Sharma et al. (2021) |
|  | 3.5–5.7 mg kg^-1^ (*C. reticulatum*) | Sharma et al. (2021) |
|  | 6.9–8 mg kg^-1^ (*C. yamashitae*) | Sharma et al. (2021) |
|  | 6.38 mg 100 g^-1^ | Fayaz et al. (2022) |
| Manganese | 18–48.2 μg g^1^ | Ereifej et al. (2001) |
|  | 1.2–2.3 mg kg^-1^ | Zia-Ul-Haq et al. (2007) |
|  | 1.31–3.69 mg 100 g^-1^ | Kaur et al. (2019) |
|  | 31.2–49.2 mg kg^-1^ (cultivated chickpea) | Sharma et al. (2021) |
|  | 58.7–65.1 mg kg^-1^ (*C. bijugum*) | Sharma et al. (2021) |
|  | 51.3–64.4 mg kg^-1^ (*C.chorassanicum*) | Sharma et al. (2021) |
|  | 18.5–20.8 mg kg^-1^ (*C. cuneatum*) | Sharma et al. (2021) |
|  | 23.9–35.9 mg kg^-1^ (*C. echinospermum*) | Sharma et al. (2021) |
|  | 77.3–99.4 mg kg^-1^ (*C. judaicum*) | Sharma et al. (2021) |
|  | 140.3–191.8 mg kg^-1^ (*C. pinnatifidum*) | Sharma et al. (2021) |
|  | 30.7–45.8 mg kg^-1^ (*C. reticulatum*) | Sharma et al. (2021) |
|  | 57.2–66.8 mg kg^-1^ (*C. yamashitae*) | Sharma et al. (2021) |
|  | 2.7. mg 100 g^-1^ | Fayaz et al. (2022) |
|  | 0.67–3.73 mg 100 g^-1^ | Roorkiwal et al. (2022) |
| Magnesium | 189.4 mg 100 g^-1^ | Patane (2006) |
|  | 1.4 –1.9 mg g^1^ | Ereifej et al. (2001) |
|  | 4.3–5 mg 100 g^-1^ | Zia-Ul-Haq et al. (2007) |
|  | 1.6–1.9 g kg^1^ | Ray et al. (2014) |
|  | 125.1–158.7 mg 100 g^-1^ | Thavarajah and Thavarajah (2012) |
|  | 156.91 mg 100 g^-1^ | Marioli Nobile et al. (2013) |
|  | 71.96–187.86 mg 100 g^-1^ | Kaur et al. (2019) |
|  | 1.21–1.59 mg kg^-1^ (cultivated chickpea) | Sharma et al. (2021) |
|  | 1.55–1.88 mg kg^-1^ (*C. bijugum*) | Sharma et al. (2021) |
|  | 1.60–1.71 mg kg^-1^ (*C.chorassanicum*) | Sharma et al. (2021) |
|  | 1.41–1.48 mg kg^-1^ (*C. cuneatum*) | Sharma et al. (2021) |
|  | 1.31–1.43 mg kg^-1^ (*C. echinospermum*) | Sharma et al. (2021) |
|  | 1.76–1.87 mg kg^-1^ (*C. judaicum*) | Sharma et al. (2021) |
|  | 1.43–1.97 mg kg^-1^ (*C. pinnatifidum*) | Sharma et al. (2021) |
|  | 1.56–2.01 mg kg^-1^ (*C. reticulatum*) | Sharma et al. (2021) |
|  | 1.22–1.28 mg kg^-1^ (*C. yamashitae*) | Sharma et al. (2021) |
|  | 64.08–134.57 mg 100 g^-1^ | Roorkiwal et al. (2022) |
|  | 197.7–230.5 mg 100 g^-1^ | Xiao et al. (2023) |
| Selenium | 15.3–56.3 μg 100 g^-1^ | Thavarajah and Thavarajah (2012) |
| Potassium | 1318.7 mg 100 g^-1^ | Patane (2006) |
|  | 9.6–17.6 mg g^1^ | Ereifej et al. (2001) |
|  | 1109–1236 mg 100 g^-1^ | Zia-Ul-Haq et al. (2007) |
|  | 732.2–1125.5 mg 100 g^-1^ | Thavarajah and Thavarajah (2012) |
| Phosphorus | 1.1–4.6 mg g^1^ | Ereifej et al. (2001) |
|  | 239–263 mg kg^-1^ | Zia-Ul-Haq et al. (2007) |
|  | 2627–3703 mg kg^-1^ | Thavarajah and Thavarajah, (2012) |
|  | 156.3 mg 100 g^-1^ | Devi et al. (2023) |
| Xanthophyll | 9.0–19.7 mg 100 g^-1^ | Thavarajah and Thavarajah (2012) |
| Canthoxanthine | 21.0–67.9 mg 100 g^-1^ | Thavarajah and Thavarajah (2012) |
| β-carotene | 166–431 μg 100 g^-1^ | Thavarajah and Thavarajah (2012) |
|  | 0.5 μg g^-1^ | Ashokkumar et al. (2015) |
|  | 0.003–0.104  mg 100 g^-1^ | Roorkiwal et al. (2022) |
| Carotenoids  Total carotenoids (violaxanthin, zeaxanthin, lutein, β-cryptoxanthin, β-carotene) | 22–44 μg g^-1^ | Rezaei et al. (2016) |
|  | 10.6–40 μg g^-1^ (four parental lines), 18.46–77.63 μg g^-1^ (segregating populations) | Rezaei et al. (2019) |
| Gamma tocopherol | 15.34 and 42.09 mg kg^-1^ | Gül et al. (2008) |
| Alpha tocopherol | 4.55 and 10.69 mg kg^-1^ | Gül et al. (2008) |
| Lutein | 8.2 μg g^-1^ | Ashokkumar et al. (2015) |
| Zeaxanthin | 6.2 μg g^-1^ | Ashokkumar et al. (2015) |
| β-cryptoxanthin | 0.1 μg g^-1^ | Ashokkumar et al. (2015) |
| Violaxanthin | 0.1 μg g^-1^ | Ashokkumar et al. (2015) |
| Folate | 351–589 μg 100 g^-1^ | Jha et al. (2015) |
|  | 0.413–6.537  mg kg^-1^ | Roorkiwal et al. (2022) |
| Vitamin B1 | 0.31–0.36 mg 100 g^-1^ | Xiao et al. (2023) |
| Phenols | 41.78 mg kg^-1^ (hexane extract) chickpea seed | Tarzi et al. (2012) |
|  | 39.2 mg kg^-1^ (methanol extract) chickpea seed | Tarzi et al. (2012) |
|  | 126 mg kg^-1^ (acetone extract) chickpea seed | Tarzi et al. (2012) |
|  | 66.90 mg kg^-1^ (hexane extract) germinated chickpea seed | Tarzi et al. (2012) |
|  | 75.60 mg kg^-1^ (methanol extract) germinated chickpea seed | Tarzi et al. (2012) |
|  | 193.70 mg kg^-1^ (acetone extract) germinated chickpea seed | Tarzi et al. (2012) |
| Phenolic compounds | 17.3–31.5 mg gallic acid equivalents (GAE) 100 g^-1^ | de Camargo et al. (2022) |
|  | 0.15–0.81 mg g^-1^ | Bhagyawant et al. (2018) |
|  | 217.69 mg GAE 100 g^-1^ | Domínguez et al. (2016) |
|  | 97–201 mg GAE 100 g^-1^ | Domínguez-Arispuro et al. (2018) |
| Flavonoids | 122.61 mg quercetin equivalents (QE) 100 g^-1^ | Domínguez et al. (2016) |
|  | 54–130 mg QE 100 g^-1^ | Domínguez-Arispuro et al. (2018) |
|  | 0.04–1.57 mg g^-1^ | Bhagyawant et al. (2018) |
| DPPH radical scavenging activity | 26.74–49.11% | Bhagyawant et al. (2018) |
| Antioxidant activity | 5707–14361 μmol TE100 g^-1^ | Domínguez-Arispuro et al. (2018) |
| Tannin | 0.232–189.63 mg g^-1^ | Bhagyawant et al. (2018) |
|  | 0.90 % | Patane (2006) |
|  | 740–763 mg 100 g^-1^ | Zia-Ul-Haq et al. (2007) |
| Phytic acid | 0.009–4.06 mg g^1^ | Bhagyawant et al. (2018) |
|  | 138–171 mg 100 g^-1^ | Zia-Ul-Haq et al. (2007) |
|  | 5.8–13.6 mg g^-1^ | Thavarajah and Thavarajah (2012) |
|  | 2.07–19.38 mg g^-1^ | Roorkiwal et al. (2022) |
| Lectin | 0.07–330.32 HU mg^-1^ | Bhagyawant et al. (2018) |
| Isoflavones (daidzin, biochanin A, genistin, troxerutin, isorhamne- tin, astilbin, L-epicatechin, astragalin, acacetin, hyperoside, and myricitrin |  | Xiao et al. (2023) |
| Isoflavones | 3078 ± 372 μg kg^-1^ | Konar et al. (2022) |
|  | ≈1282 mg 100 g^-1^ | Arora et al. (2023) |
